# A mouse model of renal fibrosis to overcome the technical variability in ischaemia/reperfusion injury among operators

**DOI:** 10.1038/s41598-019-46994-z

**Published:** 2019-07-18

**Authors:** Yu Guan, Daisuke Nakano, Yifan Zhang, Lei Li, Ye Tian, Akira Nishiyama

**Affiliations:** 1 Department of Pharmacology, Kagawa University Medical School, Kagawa, Japan; 2 Department of Urology, Beijing Friendship Hospital, Capital Medical University, Beijing, China; 3 Department of No.2 Orthopedics, Shijiazhuang City No.1 Hospital, Shijiazhuang, Hebei China

**Keywords:** Experimental models of disease, Renal fibrosis

## Abstract

The ischaemia-reperfusion (I/R) model is a widely used model of acute kidney injury (AKI) and renal fibrosis. However, the ischaemia duration that is long enough to cause broad fibrosis shows that a high mortality rate and a short ischaemia duration does not cause fibrosis, resulting in a large variation in fibrosis progression in this experimental model. Inter-operator variation occurs for I/R injury severity because the I/R procedure is complex, which results in poor reproducibility of subsequent fibrosis in the model. In the present study, we developed a renal fibrosis model in which the fibrosis progression for 8 weeks is predictable within 8 days. Three operators independently performed I/R followed by uninephrectomy at day 7 in mice. The aim was to create a model that would show a blood urea nitrogen (BUN) level >100 mg/dL at day 8 after I/R (day 1 after uninephrectomy). Although the ischaemia duration to satisfy this BUN criterion differed among operators, the mice developed anaemia, polyuria, and fibrosis in a similar manner under the same BUN criterion with a low mortality rate. Interstitial fibrosis had developed at week 8, which was strongly correlated with the BUN at day 8. This protocol allows operators to adjust the ischaemia duration based on the BUN criterion and to separate mice into the desired number of groups based on the BUN to study interventions against renal fibrosis.

## Introduction

Renal fibrosis is a common characteristic of chronic kidney disease (CKD)^1^. Acute kidney injury (AKI) becomes problematic because of its high mortality rate and lack of effective treatment^2,3^, and because of the sustained or progressive decline of renal function after the acute phase^4–6^. For example, AKI induced by ischaemia–reperfusion (I/R) stimulates excessive production and deposition of extracellular matrix proteins that can affect the long-term outcome of renal fibrosis and development of CKD and end-stage renal disease^7–9^.

In basic research, I/R kidney injury is widely used to produce renal fibrosis, and several I/R models are currently in use. The bilateral I/R model may be the closest model to clinical AKI that occurs after major cardiovascular surgery^10^; however, the ischaemic duration that is long enough to cause fibrosis development often kills the animals, and surviving animals that are potentially ‘less severe’ must then be used in these experiments. Additionally, the AKI severity could vary between the right and left kidneys in this model, and the histopathological damage to one kidney does not always reflect the renal function decline. Some groups use a unilateral I/R model after uninephrectomy (UNX) with or without a recovery period^11^. This model also has a high mortality rate. Another model is a unilateral I/R with a contralateral intact kidney^12^. This model overcomes the mortality problem, but the animals do not show a decline in renal function because the contralateral kidney compensates for the damaged kidney. The I/R kidney also shows less efficient repair after AKI compared with the above two models, resulting in shrinkage of the I/R kidney. Thus, all three of the aforementioned models share the same problem. There are few methods that estimate how much fibrosis the animals have developed or will develop before harvesting the kidney for histopathological analysis. This point makes grouping difficult when the researchers design a study to estimate the effects of specific treatments that are initiated after the onset of AKI. Measuring blood urea nitrogen (BUN) or serum creatinine during AKI may provide a rationale for the grouping using the bilateral I/R or unilateral I/R model after UNX; however, death resulting from AKI during the experimental period and eliminating the data from an animal that has died could disrupt the balance of animals within experimental groups.

Another problem with the I/R model is that there is large inter-laboratory/inter-operator variability in the AKI severity. For example, the duration of ischaemia required to increase the serum creatinine level by 1.0 mg/dL within 1 day varies widely among laboratories. This variability arises from differences in the details regarding how the I/R procedure is performed, including animal age and sex, anaesthetic use, body temperature maintenance, surgical invasion, and how precisely the renal pedicle is dissociated for clamping. These problems result in poor reproducibility of fibrosis after I/R even though the I/R model is considered to be an ‘established’ experimental AKI model.

To overcome the above-mentioned problems, we suggest a model involving unilateral I/R followed by contralateral UNX 7 days later^13^ and measurement of BUN levels on day 1 after UNX. We propose that the fibrosis development using our model is predictable within 8 days after I/R. To confirm the inter-operator variation, three operators independently performed I/R and UNX. We also assessed how the risk factors AKI, age^14,15^, and hyperglycaemia^16,17^ affect fibrosis development.

## Materials and Methods

### Materials

All chemicals with no specified manufacturer were purchased from Sigma (St. Louis, MO, USA) or Wako (Osaka, Japan).

### Animals

Male 5-week-old C57BL/6J mice and male 8-week-old KKAy mice were purchased from Clea Japan (Tokyo, Japan). Old animals were male C57BL/6J mice that were maintained in our facility at 25 °C and a 12-h/12-h light/dark cycle. All experiments were approved by the Institutional Animal Care and Use Committee of Kagawa University and followed standard guidelines for the humane care and use of animals in scientific research.

**Figure 1:**
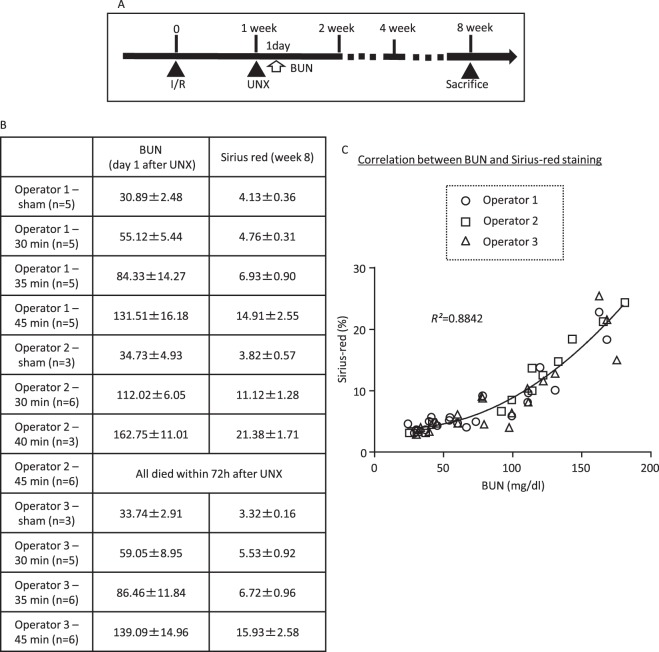
Assessment of inter-operator differences. (**A**) Schematic of the experiment schedule. (**B**) Average BUN values at day 8 and Sirius red staining at week 8 after I/R in the mice that were subjected to I/R and UNX by three independent operators. Noted that the BUN and Sirius red values of the mice received 40-min ischemia by the operator 2 are expressed as mean values ± standard errors of the means in 3 survived mice (3 of 6 died). (**C**) Correlation between BUN levels and Sirius red staining. The data from each operator were plotted independently.

To investigate the influence of factors that potentially affect the outcome, experiments using three protocols were performed, as described below. Isoflurane (1–1.5%) was used for the anaesthetics, and murine rectal temperature was maintained at 37 °C using a heat pad (BWT100A, Bio Research Center, Nagoya, Japan) during anaesthesia. All mice received acetaminophen (3 mM in drinking water) for one day after each surgery. Study 1 (ischaemic duration): C57BL/6J mice (6 weeks old, 18–22 g body weight) were separated into five groups as follows: (1) Sham surgery and then contralateral UNX 1 week later; (2) 30-min I/R and then contralateral UNX 1 week later; (3) 35-min I/R and then contralateral UNX 1 week later; (4) 40-min I/R and then contralateral UNX 1 week later; and (5) 45-min I/R and then contralateral UNX 1 week later. Unilateral renal ischaemia was performed on the left kidney via flank incision and the right kidney was removed 1 week later (Fig. 1A). To compare the difference in mortality rate upon the order of I/R and UNX, separate groups of mice subjected to UNX and then 30- or 45-min I/R with and without treatment with heparin (100 U/kg) and saline (0.5 mL) intraperitoneally in prior to I/R. Study 2 (age): C57BL/6J mice were separated into two groups as follows: (1) Young mice (6 weeks old) were subjected to 30-min I/R and then contralateral UNX 1 week later; and (2) Old mice (18 months old) were subjected to 30-min I/R and then contralateral UNX 1 week later. Study 3 (hyperglycaemia): 6-week-old C57BL/6J mice were divided into two groups as follows: (1) vehicle; and (2) streptozotocin (STZ; 50 mg/kg/day on the first day and then four injections at 10 mg/kg/day, intraperitoneally [i.p.]). Two weeks later, mice were subjected to 45-min I/R and then contralateral UNX 1 week later. To investigate the effects of anti-diabetic therapy, 9-week-old type 2 diabetic KKAy mice (35–40 g body weight) were divided into two groups as follows: (1) vehicle; and (2) luseogliflozin (a sodium glucose cotransporter 2 inhibitor as an antihyperglycemic drug, 30 mg/kg/day, orally [p.o.], before and 7 days after I/R). KKAy mice develop obesity, hyperglycaemia, hyperinsulinemia, and diabetic kidney disease^18,19^. Mice were subjected to 60-min I/R and then contralateral UNX 1 week later. Luseogliflozin was provided by Taisho-Toyama Pharma Inc. (Tokyo, Japan).

Blood samples were collected into capillary tubes from the saphenous vein to measure the haematocrit and BUN. The mice were individually housed in metabolic cages for 24 h for urine collection after an overnight acclimatisation. Blood collections after the I/R or UNX were performed at 24 hours after each procedure, and then mice were put in the metabolic cages for acclimatisation. Kidney samples were collected on the final day of each protocol.

### BUN level assay

BUN and creatinine levels were measured using commercially available assay kits (Urea Nitrogen B Test for BUN and LabAssay^TM^ Creatinine for creatinine, both from Wako, Osaka, Japan).

### Histology

The kidney was perfused with cold PBS and soaked in 4% paraformaldehyde (pH 7.4) at 4 °C for 24 h, then embedding in paraffin. Paraffin block samples were cut into 2-μm sections (three sections per mouse for each staining, 200 µm apart), and Sirius red and PAS staining were performed. Renal histological changes were observed using an optical microscope (BX-51/DP-72; Olympus, Tokyo, Japan). Sirius red-positive area was semi-quantitively determined by a point-counting technique with a grid containing 750 points (25 × 30). Five fields per section were counted and the results were expressed as a percentage of the total number of grid points. The glomerular sclerotic area was determined using ImageJ software (National Institutes of Health, Bethesda, MD, USA) and positively stained areas were analysed. All analyses were performed on blinded slides.

### Real-time PCR

A piece of renal cortical tissue was stored in RNA-later (Sigma) overnight at 4 °C. We used 7300 Fast Real-Time PCR System (Applied Biosystems, Waltham, MA, USA) to measure 18S, transforming growth factor (TGF)-β, and tumour necrosis factor (TNF)-α mRNA levels. The mouse primer sequences (forward and reverse) were as follow: 18S: 5′-GTAACCCGTTGAACCCCATT-3′, 5′-CCATCCAATCGGTAGTAGCG-3′; TGF-β: 5′-GCTGCTGACCCCCACTGATA-3′, 5′- ACAAGAGCAGTGAGCGCTGAA-3′; and TNF-α: 5′-AGCCTGTAGCCACGTCGTA-3′, 5′-TGGCACCACTAGTTGGTTGTCT-3′. Relative mRNA levels were determined using the 2^−ΔΔCt^ method. The ddCt value was calculated using data from the normal control group.

### Data and statistical analysis

Results are expressed as means ± standard errors of the mean. An *a priori* power analysis was not performed for this study. Instead we used sample sizes that previous studies indicated were sufficient to identify biologically meaningful differences. Statistical significance was assessed using a one-way analysis of variance (ANOVA) followed by Tukey’s multiple comparison test. Student’s *t*-tests were performed to compare the experimental means between two individual groups. Values of P < 0.05 were considered to be statistically significant.

## Results

### Effect of ischaemia duration on renal fibrotic outcome

Three operators who had been trained in I/R surgery more than 3 years before the experiment performed the I/R and subsequent UNX surgery in mice (Fig. 1A). Fibrosis was analysed using Sirius red staining at week 8 after I/R. In the first set of experiments, each operator performed 30-min I/R and then UNX. Operators 1 and 3 produced models that showed a similar BUN gain and polyuria after UNX, whereas Operator 2 produced the most severe BUN gain and polyuria (Supplementary Fig. S1). The model created by Operators 1 and 3 showed no decrease in haematocrit, whereas the model created by Operator 2 showed a decrease in this parameter. In the second set, Operators 1 and 3 additionally performed a sham operation, and 35- or 45-min I/R, and Operator 2 additionally performed 40- or 45-min I/R. The BUN measured on day 1 after UNX (day 8 after I/R) was increased in all I/R groups (Fig. 1B). Operators 1 and 3 produced I/R models that showed a similar BUN gain after UNX, whereas Operator 2 produced the most severe BUN gain compared with the other operators. Half of the mice (3 of 6) died of AKI within 1 day after UNX but before blood sampling in the 40-min I/R group by Operator 2, and the mice that survived this period lived until week 8. Three of the six mice subjected to 45-min I/R by Operator 2 died within 1 day after UNX, and the remaining three mice died within 3 days. No animal died in Operator 1 and 3’s groups in the I/R and subsequent UNX protocol, while the UNX and subsequent I/R protocol showed a certain rate of mortality in all Operators’ groups (Supplementary Fig. S2). There was a strong correlation (r^2^ = 0.8842) between the BUN at day 8 after I/R (day 1 after UNX) and Sirius red staining at week 8 after I/R (Fig. 1C). A quadratic approximation indicated that the kidney developed fibrosis, especially when BUN was over 100 mg/dL.

**Figure 2:**
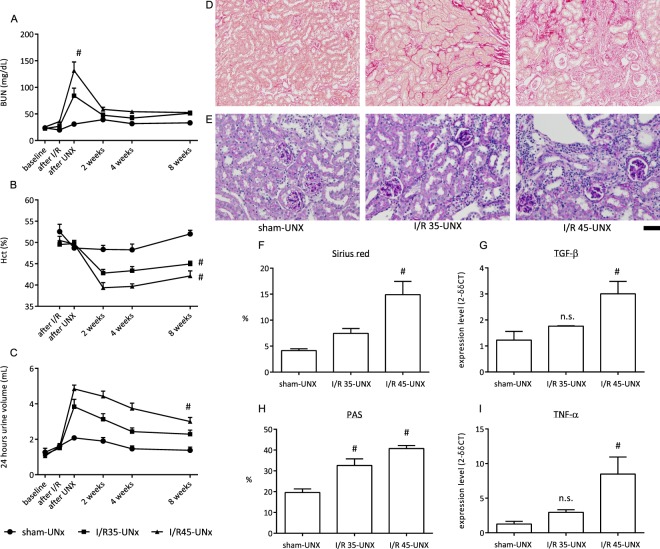
Assessment of differences in ischaemia duration. Time course changes in BUN (**A**), haematocrit (**B**), and urine volume (**C**) in mice that were subjected to either sham procedures, 35-min ischaemia/reperfusion and then uninephrectomy 1 week later (I/R35-UNX), or 45-min ischaemia/reperfusion and then uninephrectomy 1 week later (I/R45-UNX) (n = 5). Blood sampling for the “after I/R” or “UNX” was performed at 24 hours after each procedure. (**D**,**E**) Representative images of Sirius red and PAS staining in each group. Scale bar = 100 µm for Sirius red and 50 µm for PAS. (**F**) Semi-quantitative analysis of Sirius red staining in the renal cortex. (**G**) Quantification of TGF-β expression in the kidney. (**H**) Semi-quantitative analysis of PAS staining in the renal cortex. (**I**) Quantification of TNF-α expression in the kidney. ^#^p < 0.05 vs. I/R35-UNX.

**Figure 3:**
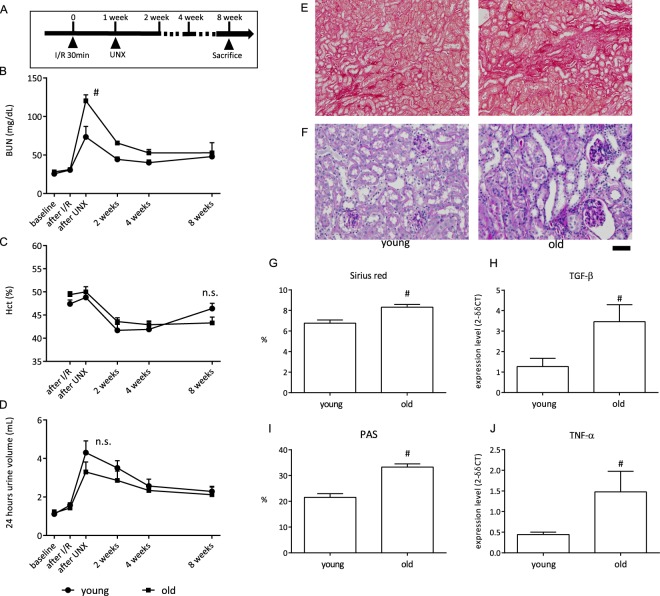
Assessment of influence by aging. (**A**) Schematic of the experiment schedule. (**B–D**) Time course changes in BUN (**B**), haematocrit (Hct) (**C**), and 24-h urine volume (**D**) in the young and old mice that were subjected to 30-min ischaemia/reperfusion (I/R) and then uninephrectomy (UNX) 1 week later (n = 6). Blood sampling for the “after I/R” or “UNX” was performed at 24 hours after each procedure. (**E**,**F**) Representative images of Sirius red and PAS staining in each group. Scale bar = 100 µm for Sirius red and 50 µm for PAS. (**G**) Semi-quantitative analysis of Sirius red staining in the renal cortex. (**H**) Quantification of TGF-β expression in the kidney. (**I**) Semi-quantitative analysis of PAS staining in the renal cortex. (**J**) Quantification of TNF-α expression in the kidney. ^#^p < 0.05 vs. young group; ns, no difference.

The importance of ischaemia duration on the development of renal fibrosis is shown in Fig. 2 using the data from a set of mice that were subjected to I/R by Operator 1. Mice were subjected to sham or 35- or 45-min I/R followed by UNX. Mice that were subjected to I/R showed an increased BUN at 1 day after UNX compared with that before UNX (Fig. 2A). The BUN was significantly higher in the 45-min I/R group compared with the 35-min I/R group. Anaemia, which is a symptom of CKD, was more severe in the 45-min I/R mice compared with 35-min I/R mice (Fig. 2B). Polyuria occurred in the I/R groups and it was greater in the 45-min I/R mice compared with the 35-min I/R mice (Fig. 2C). The creatinine clearance was significantly reduced in the 45-min I/R group compared with the sham group, but not in the 35-min I/R group (sham, 0.123 ± 0.008 mL/min; I/R 35-min-UNX, 0.094 ± 0.013 mL/min; I/R 45-min-UNX, 0.069 ± 0.010 mL/min, p < 0.05). Fibrotic tissue deposition and mesangial expansion were analysed using Sirius red (Fig. 2D,F) and PAS (Fig. 3B,E) staining, respectively, at 8 weeks after I/R. Sirius red staining showed that fibrosis development was related to the duration of ischaemia (Fig. 2E,H). PAS staining showed that glomerulosclerotic changes also developed in an ischaemia duration-dependent manner. Consistent with the Sirius red staining results, TGF-β mRNA expression (Fig. 2G) was also higher in the 45-min I/R group compared with the 35-min I/R group. As shown by control haematoxylin staining during PAS staining, massive interstitial leukocyte infiltration occurred in the 45-min I/R group (Fig. 2E), and TNF-α mRNA expression (Fig. 2I) was higher in the 45-min I/R group compared with the 35-min I/R group.

### Effect of age on renal fibrotic outcome

The importance of age on renal fibrosis development is shown in Fig. 3. Operator 1 performed 30-min I/R followed by UNX on young (6 weeks old) and old (18 months old) mice (Fig. 3A). Ischaemia duration was shortened to reduce the mortality rate in the old mice. Eight days later (1 day after UNX), BUN was increased significantly more in the old mice compared with that in the young mice (Fig. 3B). Anaemia occurred by I/R and UNX, and there was no statistical difference between young and old mice (Fig. 3C). Polyuria occurred in the I/R groups and no statistical difference was observed between young and old mice (Fig. 3D). Sirius red staining showed more fibrosis development in old mice (Fig. 3E,G), and PAS staining showed that glomerulosclerotic changes developed more in old mice (Fig. 3F,I). Consistent with the Sirius red staining results, TGF-β mRNA expression (Fig. 3H) was also higher in old mice compared with young mice. More interstitial leukocyte infiltration occurred in old mice (Fig. 3F) and TNF-α mRNA expression was greater in old mice compared with young mice (Fig. 3J).

### Effect of blood glucose on renal fibrotic outcome

**Figure 4:**
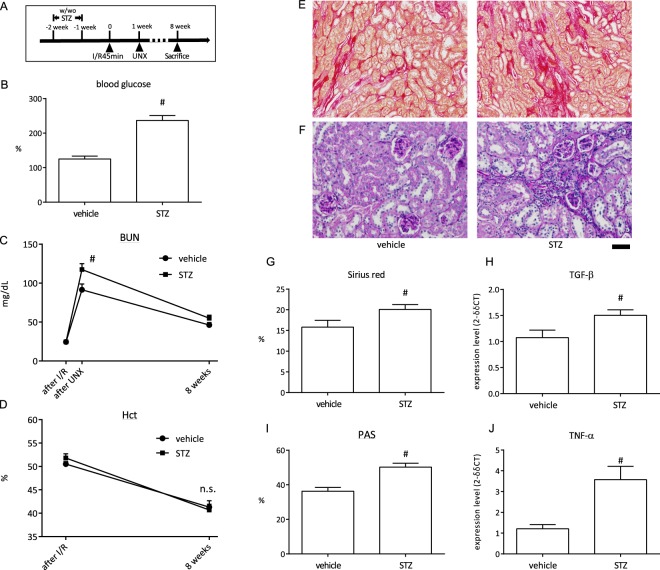
Assessment of influence by hyperglycaemia. (**A**) Schematic of the experiment schedule. (**B–D**) The level of blood glucose (**B**), BUN (**C**), and Hct (**D**) in the control and STZ-treated mice that were subjected to 45-min ischaemia/reperfusion (I/R) and then uninephrectomy (UNX) 1 week later (n = 7). Blood sampling for the “after I/R” or “UNX” was performed at 24 hours after each procedure. (**E**,**F**) Representative images of Sirius red and PAS staining in the control and STZ-treated mice. Scale bar = 100 µm for Sirius red and 50 µm for PAS. (**G**) Semi-quantitative analysis of Sirius red staining in the renal cortex. (**H**) Quantification of TGF-β expression in the kidney. (**I**) Semi-quantitative analysis of PAS staining in the renal cortex. (**J**) Quantification of TNF-α expression in the kidney. ^#^p < 0.05 vs. vehicle; ns, no difference.

The importance of blood glucose on the development of renal fibrosis was shown in Fig. 4 using data from a set of mice that were subjected to I/R by Operator 1. A high postprandial blood glucose level was induced by STZ. Mice were subjected to 45 min of I/R followed by UNX (Fig. 4A). Before I/R surgery, the blood glucose level was measured to confirm that blood glucose was high in the STZ group (Fig. 4B). The BUN level in STZ-treated mice was higher compared with vehicle-treated mice (Fig. 4C). The haematocrit (Hct) levels were lower in both groups compared with the standard values in mice, but there was no significant difference between the groups (Fig. 4D). Blood samples for BUN and Hct were collected only twice after UNX because hyperglycaemic mice are susceptible to infection. The Sirius red-positive area and TGF-β mRNA expression in STZ-treated mice were higher compared with that in normoglycemic mice (Fig. 4E,G,H). Glomerulosclerotic changes developed under hyperglycaemia (Fig. 4F,I). More interstitial leukocyte infiltration occurred in STZ mice (Fig. 4F) and TNF-α mRNA expression was higher in STZ mice compared with vehicle-treated mice (Fig. 4J).

**Figure 5:**
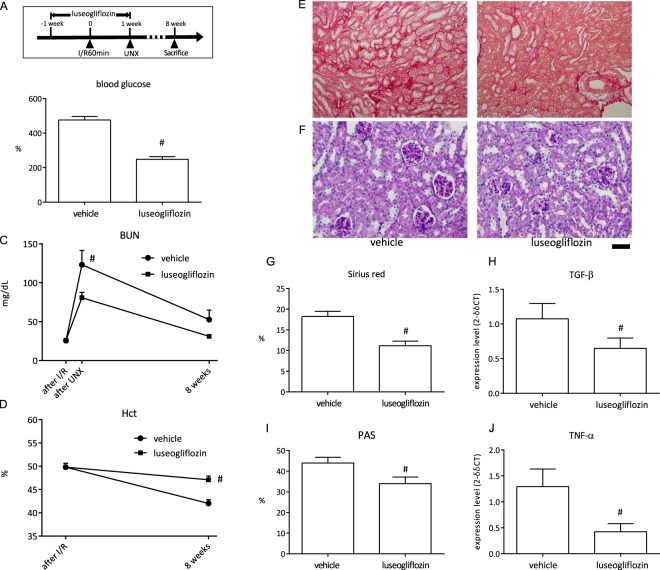
Assessment of influence by an anti-hyperglycaemic drug. (**A**) Schematic of the experiment schedule. (**B–D**) The level of blood glucose (**B**), BUN (**C**), and Hct (**D**) in the vehicle and Luseogliflozin treated KKAy mice that were subjected to 60-min ischaemia/reperfusion (I/R) and then uninephrectomy (UNX) 1 week later (n = 5). Blood sampling for the “after I/R” or “UNX” was performed at 24 hours after each procedure. (**E**,**F**) Representative images of Sirius red and PAS staining in the vehicle or luseogliflozin-treated KKAy mice. Scale bar = 100 µm for Sirius red and 50 µm for PAS. (**G**) Semi-quantitative analysis of Sirius red staining in the renal cortex. (**H**) Quantification of TGF-β expression in the kidney. (**I**) Semi-quantitative analysis of PAS staining in the renal cortex. (**J**) Quantification of TNF-α expression in the kidney. ^#^p < 0.05 vs. vehicle.

Next, we used a type 2 diabetic model, KKAy mice, and treated the KKAy mice with luseogliflozin, which is an SGLT2 inhibitor. The luseogliflozin therapy was stopped before UNX (Fig. 5A). Mice were subjected to 60-min I/R followed by UNX. A longer ischaemia protocol was required because the KKAy mice were more resistant to an increase in BUN after UNX compared with the C57Bl/6 strain. This might be also partially caused by the age of the mice when I/R surgery was performed; mice were 4 weeks older in this experiment compared with the previous protocol. The blood glucose level before I/R was lower in luseogliflozin-treated KKAy mice compared with vehicle-treated KKAy mice (Fig. 5B). The BUN level in vehicle-treated KKAy mice was higher compared with the luseogliflozin-treated KKAy mice (Fig. 5C). The Hct levels were higher in luseogliflozin-treated KKAy mice compared with vehicle-treated KKAy mice (Fig. 5D). The Sirius red-positive area and TGF-β mRNA expression in vehicle-treated KKAy mice were higher compared with those in luseogliflozin-treated KKAy mice (Fig. 5E,G,H). The PAS-positive area was smaller in luseogliflozin-treated KKAy mice compared with vehicle-treated KKAy mice (Fig. 5F,I). TNF-α mRNA expression was greater in vehicle-treated KKAy mice compared with luseogliflozin-treated mice (Fig. 5J).

### Correlation between BUN and fibrosis in animals with risk factors

**Figure 6:**
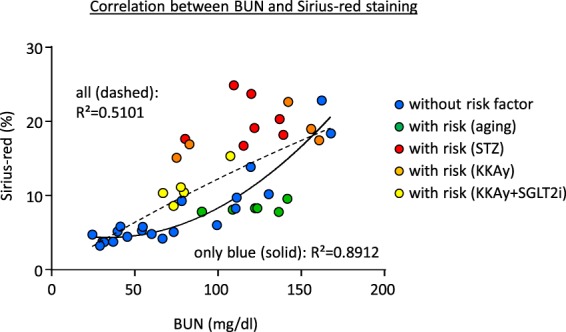
Correlation between the BUN at day 8 after I/R and Sirius red staining at week 8 after I/R. The data from each mouse with or without risk factors was plotted independently.

The association between BUN and Sirius red staining in animals with and without risk factors, which were assessed by Operator 1, is presented in Fig. 6. Most animals with diabetes showed more severe fibrosis and most of old mice showed less severe fibrosis than were expected based on the correlation curve from animals without risk factors, and the R^2^ value became smaller than the curve based only on data from animals without risk factors.

## Discussion

The current study presents a mouse model of renal fibrosis after I/R. The BUN at day 1 after UNX allows prediction of the renal fibrosis severity that will develop in the mice, and this provides a strong benefit for designing intervention studies against renal fibrosis (Supplementary Fig. S3). In the ‘classical’ model of I/R that is used to develop fibrosis, the mice need to receive strong AKI for the future development of tubulointerstitial fibrosis, which results in a high mortality rate in the acute phase. The BUN level during AKI cannot be a prediction marker because the values in the dead mice must be eliminated, which makes the number of animals as well as average BUN value in each group unequal. However, the proposed model showed a reduced mortality rate, while mice that survived for 3 days after UNX showed no mortality in the present study. Thus, using the proposed model, researchers can divide the mice into groups containing the desired number of animals based on the BUN levels and subsequently initiate the experiments. Mice that showed low BUN levels, such as below 100 mg/dL on day 1 after UNX, can be excluded from the study because fibrosis development is not expected. In the present study, I/R that was independently performed by multiple operators showed different BUN severities, as expected. However, the fibrosis severity was strongly correlated with the BUN level at day 1 after UNX in the experiments performed by all operators, and there was low inter-operator variability in fibrosis development when the groups were assessed based on the BUN level. Thus, this protocol can provide a predictable renal fibrosis mouse model with a relatively low mortality rate.

The risk factors for AKI, such as aging and diabetes, increased fibrosis development. A weak correlation was observed if the mice with risk factors were included in the analysis. Most of the mice with diabetes showed more severe fibrosis than was expected based on the correlation curve for normal mice. AKI induced by renal I/R is characterised by acute tubular necrosis and cast formation^3^. The tubules, after the acute event, initiate the recovery process during which the surviving tubular cells proliferate and heal the wounds. Incomplete recovery results in accelerating renal fibrotic changes^20^. We reported previously that hyperglycaemia contributes to the development of cellular senescence in the diabetic mouse kidney^21^. It is likely that recovery is less efficient in animals with these AKI risk factors, and this resulted in the unpredictable increase of renal fibrosis. Anti-hyperglycaemic treatment with luseogliflozin during subclinical AKI suppressed the increase in BUN, anaemia, fibrosis, and glomerulosclerosis, suggesting that suppressing hyperglycaemia and reducing the risk factors for AKI could ameliorate the fibrotic outcome. However, most of the old mice showed less severe fibrosis than was expected based on the correlation curve for normal mice. It has been reported that tubular cells in old mice are prone to senescence, and the old mice showed a slower recovery after renal I/R injury^22,23^. Therefore, this result was unexpected. One possible explanation is the sensitivity of BUN to renal I/R injury in old mice. It is well known that older mice are more easily dehydrated, and that dehydration induces an increase in BUN.

Renal fibrosis is a critical target for drug development against renal diseases. Our model in the present study can allow laboratories/operators to use different ischaemia durations for preparing a fibrosis model among normal mice after a preliminary experiment for finding the ischaemia duration–BUN relationship for each laboratory environment and operator skill. However, we cannot identify any model that has the same purpose and that also has AKI risk factors. A significant percentage (or most) of AKI patients have risk factor(s) and these risk factors will become targets of future drug development research, either for prophylactic or treatment purposes. Thus, the next challenge should be to develop a renal fibrosis model that can allow animals with risk factors to be divided into equal groups after the onset of ischaemic events.

## Supplementary information

supplementary figures
