# Supplementary material for: A mouse model of renal fibrosis to overcome the technical variability in ischaemia/reperfusion injury among operators
Source: Sci Rep. 2019 Jul 18;9:10435. doi: 10.1038/s41598-019-46994-z (PMC6639321; doi:10.1038/s41598-019-46994-z)
Supplement: Supplementary file 1 — supplementary figures [file 41598_2019_46994_MOESM1_ESM.pdf]

Supplementary information

A mouse model of renal fibrosis to overcome the technical variability in ischaemia/reperfusion injury among operators

Yu Guan[1,2], Daisuke Nakano[1], Yifan Zhang[1,3], Lei Li[1], Ye Tian[2], Akira Nishiyama[1]

[1]Department of Pharmacology, Kagawa University Medical School, Kagawa, Japan

[2]Department of Urology, Beijing Friendship Hospital, Capital Medical University, Beijing, China

[3]Department of No.2 Orthopedics, Shijiazhuang City No.1 Hospital, Shijiazhuang, Hebei, China

Running title:

A renal fibrosis mouse model after I/R

Correspondence:

Daisuke Nakano, PhD, Department of Pharmacology, Faculty of Medicine, Kagawa University, 1750-1 Ikenobe, Miki, Kita, Kagawa 761-0793, Japan

Tel: +81 (87) 891-2125; Fax: +81 (87) 891-2126

E-mail: dnakano@med.kagawa-u.ac.jp

Supplementary Figure S1. Assessment of inter-operator difference on renal function after 30-min ischaemia and reperfusion (I/R) and subsequent uninephrectomy (UNX). Time course changes in the BUN level (A) and urine volume (B) (n=5–6), and haematocrit level (n=3–10) at week 8 (C) after I/R in mice that were subjected to either sham procedures, or 30-min I/R and then UNX 1 week later. Haematocrit levels of mice subjected 35- or 45-min I/R and then UNX by Operator 1, which are also presented in Figure 2 in the main text, were used as the reference.

Supplementary Figure S2. Mortality rate of mice in two different protocols used for ischaemia/reperfusion (I/R) models. The left table describes the experimental settings and mortality rates of mice subjected to uninephrectomy (UNX) and then ischaemia/reperfusion with and without treatment with heparin and saline. Eight of nine protocols performed by Operator 1, 2, or 3 resulted in mouse mortality within 24 hours after the I/R. The right table shows the mortality rate of the current protocol. Forty- or 45-min ischaemia and reperfusion and then UNX by Operator 2 showed 50% of mortality rate but the other protocols showed 0% of mortality rate. *1, The BUN data was published in Zhang et al. Kidney Int. 2018; 94(3):524-535. *2, The BUN data was published in Zhang et al. Journal of Urology and Nephrology Open Access. 2016; 2(1): 1-5.

Supplementary Figure S3. The classical ischaemia/reperfusion (I/R) model for fibrosis induces a high mortality rate in the acute phase. The experimental group could be separated based on body weight or BUN level during AKI; however, the values in the dead mice must be discarded, which disrupts the balance between the groups. The proposed model showed a reduced mortality rate, and it allows researchers to divide the

mice into groups based on the BUN levels after uninephrectomy (UNX) and subsequently initiate the experiments. Mice that showed low BUN levels, such as below 100 mg/dL at day 1 after UNX, can be excluded from the study because fibrosis development is not expected. Then, if the candidate drug against renal fibrosis really works, researchers will see a reduction in fibrosis at week 8.

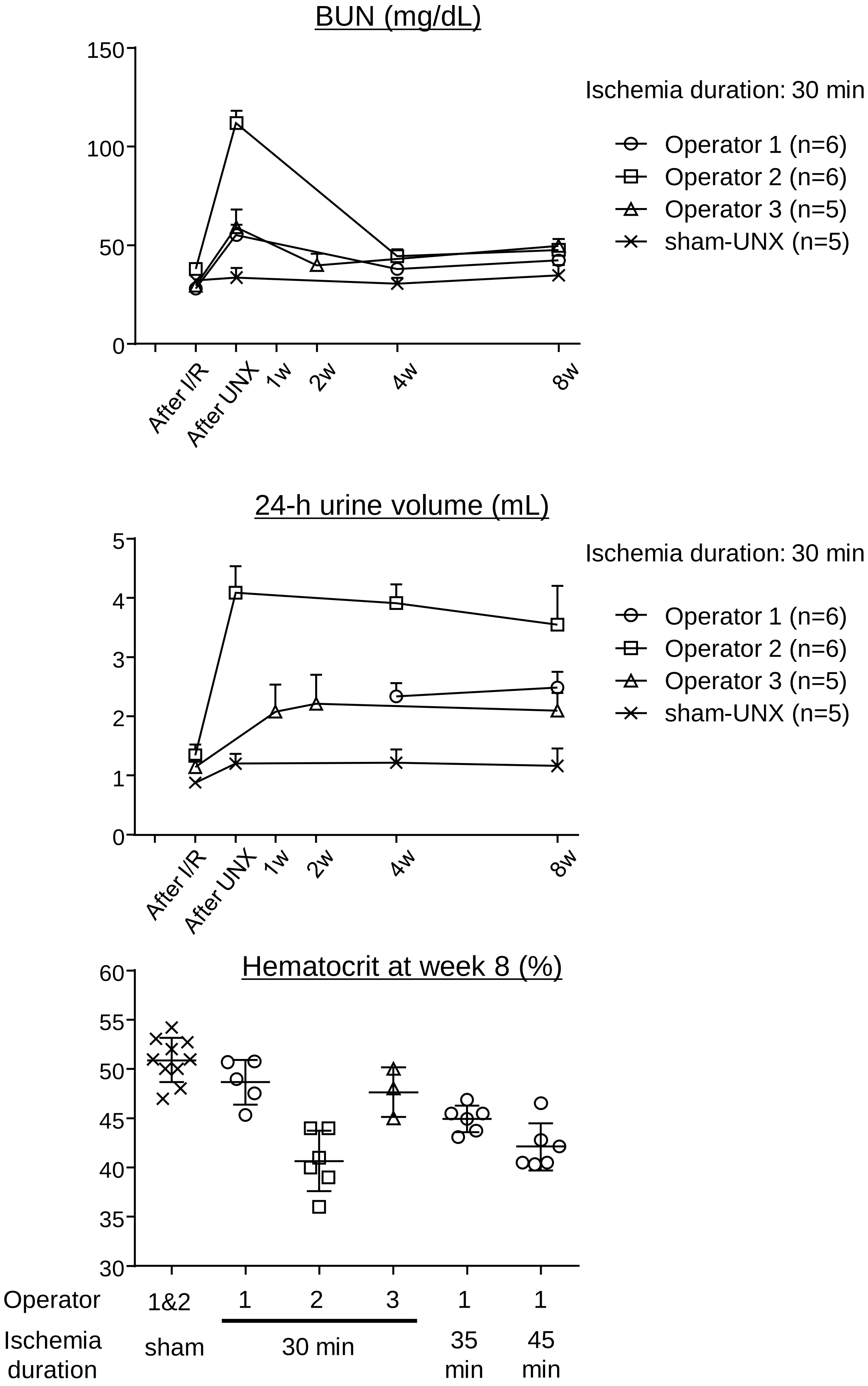

BUN (mg/dL)
150
100
50
0
After I/R
After UNX
1w
2w
4w
8w
Ischemia duration: 30 min
Operator 1 (n=6)
Operator 2 (n=6)
Operator 3 (n=5)
sham-UNX (n=5)
24-h urine volume (mL)
5
4
3
2
1
0
After I/R
After UNX
1w
2w
4w
8w
Ischemia duration: 30 min
Operator 1 (n=6)
Operator 2 (n=6)
Operator 3 (n=5)
sham-UNX (n=5)
Hematocrit at week 8 (%)
60
55
50
45
40
35
30
Operator
1&2
1
2
3
1
1
Ischemia duration
sham
30 min
35 min
45 min

UNX-I/R

which operator	UNX	ischemia duration	treatment	mortality within 24h (%)	n	BUN of survivor at 24h (mg/dL)
operator 1	10 days before I/R	30	none	16	2 of 12	63±11
	10 days before I/R	45	none	50	4 of 8	172±13
operator 2	10 days before I/R	30	none	14	1 of 7	90±12 *[1]
	10 days before I/R	45	none	79	19 of 24	215±5
	10 days before I/R	30	heparin (100U/kg)& saline (0.5 mL)	0	0 of 7	70±12
	10 days before I/R	45	heparin (100U/kg)& saline (0.5 mL)	25	2 of 8	104±5
operator 3	10 days before I/R	30	heparin (100U/kg)& saline (0.5 mL)	22	5 of 23	133±3 *[2]
	10 days before I/R	45	heparin (100U/kg)& saline (0.5 mL)	40	16 of 40	166±5 *[2]

I/R-UNX

which operator	UNX	ischemia duration	treatment	mortality within 24h (%)	n	BUN of survivor at 24h (mg/dL)
operator 1	7 days after I/R	30	none	0	0 of 5	55±5
	7 days after I/R	35	none	0	0 of 5	84±14
	7 days after I/R	45	none	0	0 of 5	132±16
operator 2	7 days after I/R	30	none	0	0 of 6	112±6
	7 days after I/R	40	none	50	3 of 6	163±11
	7 days after I/R	45	none	50	3 of 6	no data
operator 3	7 days after I/R	30	none	0	0 of 5	59±9
	7 days after I/R	35	none	0	0 of 6	86±12
	7 days after I/R	45	none	0	0 of 6	139±15

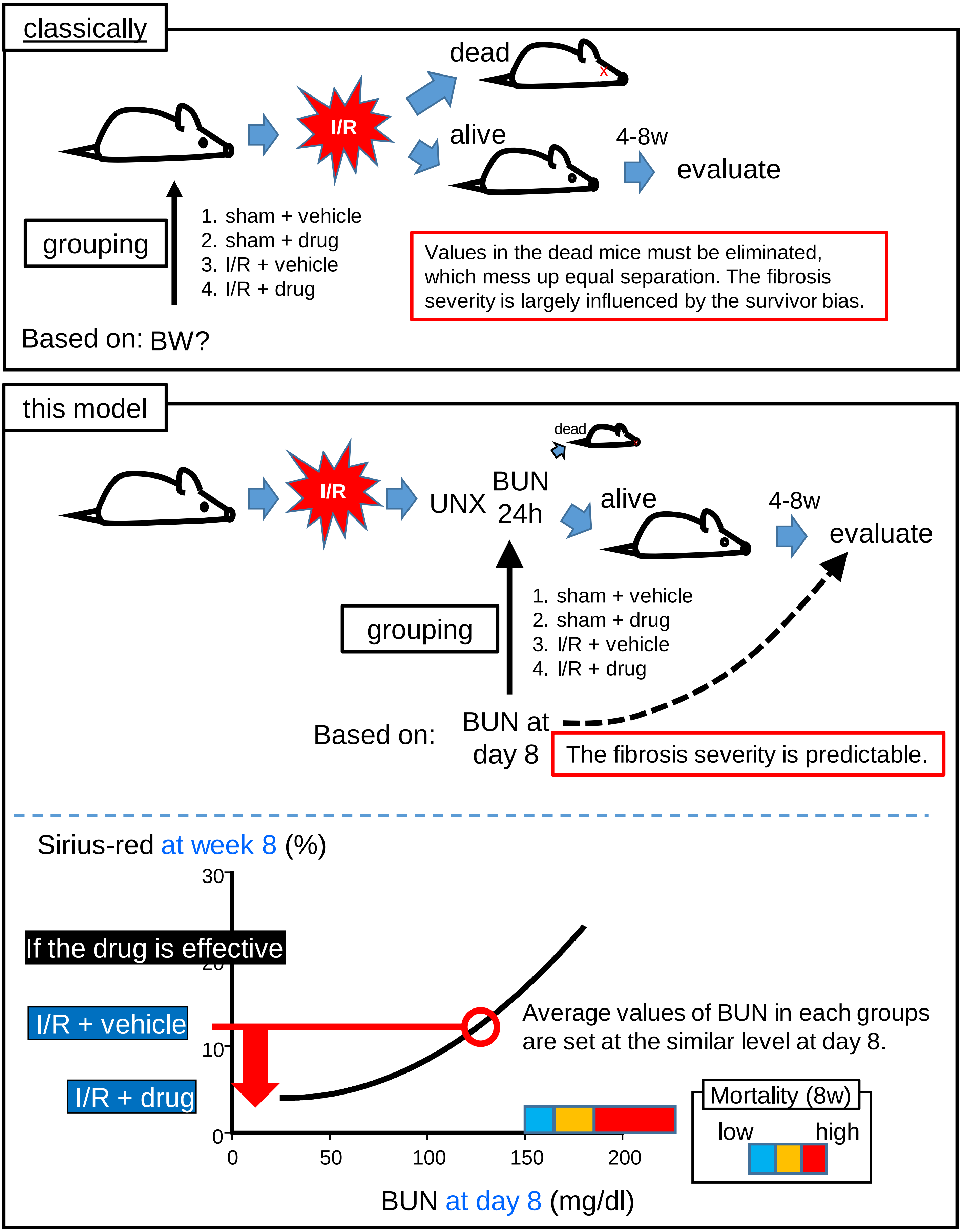
classically
dead
alive
I/R
4-8w
evaluate
grouping
1. sham + vehicle
2. sham + drug
3. I/R + vehicle
4. I/R + drug
Values in the dead mice must be eliminated, which mess up equal separation. The fibrosis severity is largely influenced by the survivor bias.
Based on: BW?
this model
I/R
UNX
BUN 24h
dead
alive
4-8w
evaluate
grouping
1. sham + vehicle
2. sham + drug
3. I/R + vehicle
4. I/R + drug
Based on:
BUN at day 8
The fibrosis severity is predictable.
Sirius-red at week 8 (%)
30
10
0
If the drug is effective
I/R + vehicle
I/R + drug
Average values of BUN in each groups are set at the similar level at day 8.
0
50
100
150
200
BUN at day 8 (mg/dl)
Mortality (8w)
low
high
